# German *Culex pipiens* biotype *molestus* and *Culex torrentium* are vector-competent for Usutu virus

**DOI:** 10.1186/s13071-020-04532-1

**Published:** 2020-12-30

**Authors:** Cora M. Holicki, Dorothee E. Scheuch, Ute Ziegler, Julia Lettow, Helge Kampen, Doreen Werner, Martin H. Groschup

**Affiliations:** 1 Institute of Novel and Emerging Infectious Diseases, Friedrich-Loeffler-Institut, Federal Research Institute for Animal Health, Greifswald-Insel Riems, Germany; 2 Institute of Infectiology, Friedrich-Loeffler-Institut, Federal Research Institute for Animal Health, Greifswald-Insel Riems, Germany; 3 Present Address: Department of Molecular Genetics and Infection Biology, University of Greifswald, Greifswald, Germany; 4 Biodiversity of Aquatic and Semiaquatic Landscape Features, Leibniz Centre for Agricultural Landscape Research, Müncheberg, Germany

**Keywords:** *Culex pipiens* biotype *molestus*, *Culex torrentium*, *Aedes aegypti*, Vector competence, Usutu virus

## Abstract

**Background:**

Usutu virus (USUV) is a rapidly spreading zoonotic arbovirus (arthropod-borne virus) and a considerable threat to the global avifauna and in isolated cases to human health. It is maintained in an enzootic cycle involving ornithophilic mosquitoes as vectors and birds as reservoir hosts. Despite massive die-offs in wild bird populations and the detection of severe neurological symptoms in infected humans, little is known about which mosquito species are involved in the propagation of USUV.

**Methods:**

In the present study, the vector competence of a German (i.e. “Central European”) and a Serbian (i.e. “Southern European”) *Culex pipiens* biotype *molestus* laboratory colony was experimentally evaluated. For comparative purposes, *Culex torrentium*, a frequent species in Northern Europe, and *Aedes aegypti*, a primarily tropical species, were also tested. Adult female mosquitoes were exposed to bovine blood spiked with USUV Africa 2 and subsequently incubated at 25 °C. After 2 to 3 weeks saliva was collected from each individual mosquito to assess the ability of a mosquito species to transmit USUV.

**Results:**

*Culex pipiens* biotype *molestus* mosquitoes originating from Germany and the Republic of Serbia and *Cx. torrentium* mosquitoes from Germany proved competent for USUV, as indicated by harboring viable virus in their saliva 21 days post infection*.* By contrast, *Ae. aegypti* mosquitoes were relatively refractory to an USUV infection, exhibiting low infection rates and lacking virus in their saliva.

**Conclusions:**

Consistent with the high prevalences and abundances of *Cx. pipiens* biotype *molestus* and *Cx. torrentium* in Central and Northern Europe, these two species have most likely played a historic role in the spread, maintenance, and introduction of USUV into Germany. Identification of the key USUV vectors enables the establishment and implementation of rigorous entomological surveillance programs and the development of effective, evidence-based vector control interventions.
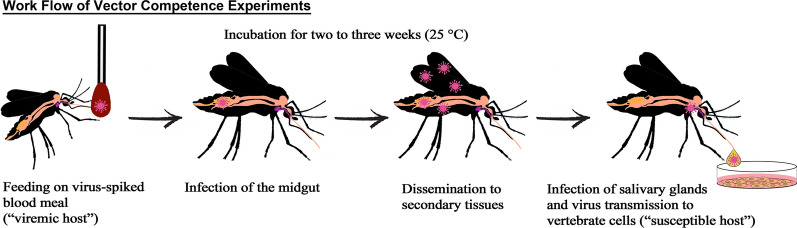

## Background

An increase in international trade and travel, combined with climate change, has led to the recent introduction and spread of arthropod-borne pathogens into Europe [1]. The (re-)emergence of arboviruses threatens human and animal health and can cause high economic losses [2]. For example, West Nile virus (WNV) can cause encephalitis or meningoencephalitis, with a possible lethal outcome in infected humans [3], and dengue and chikungunya viruses can lead to hemorrhagic and thereby sometimes fatal illnesses [4, 5].

Usutu virus (USUV; *Flaviviridae*, *Flavivirus*) is currently considered an emerging zoonotic arbovirus in Europe with an increase in registered cases and observed disease severity in both humans and animals. This flavivirus belongs to the Japanese encephalitis virus serocomplex with birds, mainly Eurasian blackbirds (*Turdus merula*), Eurasian magpies (*Pica pica*), house sparrows (*Passer domesticus*), and great grey owls (*Strix nebulosa*), as amplifying hosts [6, 7]. Susceptible avian species show mild to severe clinical signs including encephalitis, myocardial degeneration, and necrosis of the liver and spleen [8, 9]. Although USUV primarily causes a self-limiting, febrile illness with seroconversion in incidental hosts such as humans and horses, a neuroinvasive disease in humans, primarily in immunocompromised patients, should receive more attention. Recent studies even indicate that human USUV infections are not as sporadic as previously presumed [6, 10–12], with so far 49 reported acute infections over the years (1981–2018) in the Central African Republic, Burkina Faso, Italy, Croatia, Germany, France, and Austria [6].

USUV was first detected by McIntosh in 1959 in a *Culex neavei* mosquito (originally described as *Cx. univittatus*) near the Usutu River, Natal, South Africa [13]. Phylogenetic analyses suggest a minimum of three unique USUV dispersions from Africa to Europe (in the 1950s, 1980s, and 1990s). Presumably, the virus arrived along the eastern Atlantic and Black Sea/Mediterranean routes [6] with migratory birds as long-distance dispersal vehicles [14]. The first recorded outbreak of USUV in Europe occurred in 2001 in Austria among Eurasian blackbirds and great grey owls [15]. Thereupon, USUV ribonucleic acid (RNA) was retrospectively isolated from tissue samples of Eurasian blackbirds from 1996 in Italy [16]. Since then, USUV continued to colonize new ecological niches throughout Europe. In Germany, the first documented isolation of USUV was in 2010 from a pool of *Cx. pipiens* biotype *pipiens* mosquitoes collected in the city of Weinheim in the southwest of the country [17]. In the following 2 years, several epidemics in the Upper Rhine Valley resulted in substantial mortalities among the wild avifauna, primarily of the Eurasian blackbird [18]. To date, USUV has spread to the north and east of Germany, covering all German federal states, with major epidemics in 2016 and 2018 [19].

USUV is currently classified into eight genetically distinct lineages [20] depending on their geographical origin of isolation [14], of which five circulate in Germany: “Africa 2,” “Africa 3,” “Europe 2,” “Europe 3,” and “Europe 5” [8, 18–20]. The Africa 2 clade, for example, originated from South Africa and was isolated in Germany in 2015 from two carcasses of juvenile great grey owls from the Zoological Garden Berlin [8]. Till now, it is uncertain which vectors play an important role in the maintenance and spread of USUV in Germany.

USUV has been isolated from numerous mosquito species yet the primary vectors belong to the genus *Culex* [6]. This genus contains species that already are known to be fundamental in the spread of multiple other arboviruses including Sindbis virus, St. Louis encephalitis virus, Rift Valley fever phlebovirus, and WNV [21]. So far *Cx. neavei* from Senegal [22]*, Cx. pipiens* and *Cx. quinquefasciatus* from North America [23], and *Cx. pipiens* from The Netherlands [24] have proved to be highly competent for USUV under laboratory conditions. In Europe, *Cx. pipiens* is highly abundant [25] and can be subdivided into two distinct biotypes: biotype *pipiens* (Linnaeus 1758) and biotype *molestus* (Forskål 1775), which vary greatly in their physiological and behavioral characteristics [26]. In Germany, both biotypes occur in sympatry or as hybrids, and their relevance for the spread of German USUV strains must be further clarified [27]. *Culex torrentium*, another common species of temperate regions (e.g. Northern Europe), exhibits similar feeding-preferences as biotype *pipiens*, with both preferring to feed on birds, unlike the mammalophilic biotype *molestus* [26]. Primarily due to difficulties in the establishment of *Cx. torrentium* laboratory colonies, research on this species is very limited. Furthermore, there are only a few studies on the role of *Aedes* species such as *Ae. aegypti* or the invasive species *Ae. japonicus* and *Ae. albopictus* in the USUV transmission cycle [23, 28, 29]. To date, both an Italian and a North American *Ae. albopictus* population have been found to be refractory to an USUV infection [23, 28] while an *Ae. japonicus* population from The Netherlands could transmit USUV in its saliva [29].

In this research, vector competence studies were performed with German mosquito populations to better comprehend and predict the spread of enzootic USUV strains throughout Germany and of strains newly introduced into the country. Infection experiments were carried out with German *Cx. pipiens* biotype *molestus* (i.e. a mammalophilic species, possibly transmitting USUV to humans) and *Cx. torrentium* (i.e. an ornithophilic species, possibly essential for sustaining the enzootic transmission cycle between birds) to investigate their susceptibility for the USUV Africa 2 strain, “USUV-Berlin” [8]. For comparison purposes, the vector competence of “Southern European,” Serbian *Cx. pipiens* biotype *molestus*, and Malaysian *Ae. aegypti* colonies were also examined.

## Methods

### Mosquito collection, rearing, and identification

For the infection experiments, the following laboratory colonies were used: *Cx. pipiens* biotype *molestus* from the “Wendland,” Lower Saxony, Germany (established in 2012); *Cx. pipiens* biotype *molestus* from Novi Sad, the  Republic of Serbia (established in 2012); *Ae. aegypti* originally from Malaysia (Bayer CropScience, Langenfeld, Germany). All the tested mosquito colonies were reared at 24 °C ± 1 °C with a relative humidity of approximately 60–70% and a 16 h light/8 h dark photocycle. The adult mosquitoes were provided with a 5–6% sugar solution ad libitum and for egg production with bovine or chicken (EDTA or heparin) whole blood, depending on the feeding preference of the species/biotype. *Culex torrentium* females were produced from egg rafts collected in the field from different populations near Berlin and Bonn, Germany, after species identification of two to three larvae hatching from each raft by means of a real-time polymerase chain reaction (PCR) [27]. To ensure that all tested mosquito populations were free from flaviviruses prior to the experiments, individual non-engorged females per population were examined via an USUV-specific reverse transcription quantitative real-time PCR (RT-qPCR) [17] and a SYBR^®^ Green-based quantitative real-time pan-flavivirus assay [30].

### Virus strain and cultivation

An USUV Africa 2 strain was used for all infection experiments. The strain was isolated from the brain of a succumbed great grey owl in Berlin, Germany, in 2015 (GenBank accession no. KU664608) [8]. The virus was passaged twice on confluent monolayers of Vero cells (Collection of Cell Lines in Veterinary Medicine, Friedrich-Loeffler-Institut (FLI), Greifswald-Insel Riems, Germany), and cell culture supernatant was harvested 4 days post infection (dpi). The virus stock was aliquoted and stored in cryovials at – 70 °C until further use. For virus cultivation, Vero cells were maintained in minimum essential medium (MEM), supplemented with 2% fetal calf serum (FCS) and 1% antibiotics (penicillin and streptomycin; Merck, St. Louis, MO, USA). Virus was quantified by means of an endpoint dilution assay, and the virus titer was calculated with the Spearman-Karber algorithm [31]. The used stock had a titer of approximately 10^8.1^ 50% tissue culture infective dose (TCID_50_) per ml.

### Oral infection with USUV

Adult mosquitoes (3–14 days old) were sorted into groups of ten individuals under 100% carbon dioxide (CO_2_) anesthesia 1 day prior to infection. After sugar deprivation for 24–48 h, *Culex* mosquitoes were fed artificially overnight. They were exposed to cotton swab ends soaked in an infectious blood meal. *Aedes* mosquitoes were allowed to feed for 1 h on virus-spiked blood offered via a Hemotek PS5 feeder (Hemotek Ltd, Lancashire, UK), using hog gut and parafilm to seal-off the reservoirs. The blood meal consisted of 10% virus stock, 60% bovine EDTA blood, and 30% sugar solution with a stock concentration of 5–6%. To confirm that the used blood was free from WNV-specific antibodies, serum samples from the cows were examined with the ID Screen^®^ WN competition enzyme-linked immunosorbent assay (ELISA) (IDVet, Grabels, France). After the preparation of the blood meal, a remainder of it was titrated on Vero cells to calculate the exact virus titer in TCID_50_/ml, which varied between the experiments. In two of the experiments, the virus titer of the blood meal had dropped by about two logs for unknown reasons. The mosquitoes were, therefore, split into two groups, one was exposed to a high virus titer (10^7.4^ TCID_50_/ml) and the other to a low virus titer (10^5.1^ TCID_50_/ml).

Engorged mosquitoes were transferred under 100% CO_2_ sedation into chambers and kept in an incubator (climate test cabinet MKKL, Flohr Instruments, Nieuwegein, The Netherlands; dehumidifier MG50, Munters, Bedfordshire, UK) under 25 °C ± 1 °C and a relative humidity of 80–85% and offered cotton pads soaked with 5–6% sugar solution ad libitum. A minimum of one engorged female per species/population was frozen at – 70 °C to confirm virus ingestion and as a baseline for virus replication in the mosquitoes.

### Forced salivation assay

By means of forced salivation, saliva was collected after an incubation period of both 14 and 16 (14/16) or 21 days, respectively. After immobilization of the mosquitoes by removing their legs and wings under 100% CO_2_ anesthesia, their probosces were inserted into cut 10-µl filter tips filled with 10 µl of phosphate-buffered saline (PBS). Saliva was collected for half an hour, and the tip content was subsequently transferred into a 1.5-ml tube containing another 10 µl of PBS [32]. Directly thereafter, the mosquito saliva samples were inoculated onto a 96-well plate cell monolayer of Vero cells (i.e. 20 µl of each saliva solution was diluted in 150 µl of MEM + 2% FCS + 1% antibiotics). After 7 days, the cells were examined under the light microscope, fixed with 7.5% neutral buffered formalin (Carl Roth, Karlsruhe, Germany), and stained with crystal violet (Carl Roth). If the saliva appeared to contain viable and replicable virus after the 7 days (i.e. represented by a distinct cytopathogenic effect in the Vero cells), 140 µl of the cell culture supernatant was collected before fixing, and the RNA was extracted using the QIAamp Viral RNA Mini Kit (QIAGEN, Hilden, Germany) according to manufacturer’s instructions. RNA extracts, eluted in 50 µl of the elution buffer, were tested with an USUV-specific RT-qPCR assay (5 µl at a time) [17] with a standard curve running in parallel [33]. All RT-qPCR assays were completed with the AgPath-ID One-Step RT-PCR Reagents (ThermoFischer Scientific, Darmstadt, Germany) and the CFX96™ Real-Time PCR Detection System (Bio-Rad Laboratories, Feldkirchen, Germany). To confirm correct species association, mosquitoes which contained USUV in their saliva were retroactively once again identified to species and biotype level. For the sake of completeness this was not only done with the field-derived mosquitoes (i.e. positive *Cx. torrentium*) but also with the mosquitoes originating from the laboratory colonies. For molecular identification a real-time PCR assay [27] was used for the *Cx. pipiens* complex and cytochrome *c* oxidase subunit 1 barcoding [34, 35] for *Ae. aegypti.*

### Pathogen screening in mosquito bodies and legs plus wings

After saliva collection, the mosquito bodies (thorax and abdomen) and legs plus wings were stored separately in 2-ml screw cap tubes with two 3-mm steel beads, 560 µl of AVL viral lysis buffer, and carrier RNA (QIAGEN) at – 70 °C. For RNA extraction, mosquito bodies and legs plus wings were homogenized for 2 min at 30 Hz (TissueLyser II; QIAGEN). Next, insoluble debris was pelleted by centrifugation for 1 min at 13,000 rpm (5430R centrifuge; Eppendorf, Hamburg, Germany), and RNA was extracted from the supernatant with a BioSprint 96 (QIAGEN) or KingFisher Flex Purification System (ThermoFischer) using the NucleoMag VET kit (MACHEREY-NAGEL, Düren, Germany) and following the manufacturer’s instructions. RNA extracts were eluted in 100 µl elution buffer and stored at – 70 °C. RNA solutions (5 µl at a time) were tested for viral RNA, using the same USUV-specific RT-qPCR assay as above [17] with the standard curve for quantification [33] and the CFX96™ Real-Time PCR Detection System (Bio-Rad Laboratories).

### Interpretation of results

Feeding rate refers to the number of engorged females compared to the overall number of females exposed to the infectious blood meal. Survival rate at a given time point is defined as the number of live mosquitoes out of the total number of engorged females subjected to the experiment (minus the day-0 samples). Mosquito bodies, legs plus wings, and saliva samples were considered USUV-positive if they contained USUV-specific RNA (cycle threshold < 36). A minimum of two technical replicates were completed for each positive sample in the RT-qPCR assay [17]. The infection rate describes the number of infected mosquitoes (i.e. USUV-positive bodies) out of the total number of mosquitoes analyzed. To verify that USUV infection and replication in the respective species were, however, not limited to the midgut and that viral dissemination to secondary tissues took place, legs plus wings were also examined. The dissemination rate is defined as the number of USUV-positive legs plus wings samples among the infected (i.e. USUV-positive bodies) mosquitoes. Nonetheless, a mosquito species was only considered competent for USUV when viral RNA was found in its saliva. Transmission rate is measured as the number of USUV-positive saliva samples out of the number of mosquitoes with a disseminated infection (i.e., USUV-positive bodies and legs plus wings). Transmission efficiency describes the total number of USUV-positive saliva samples out of the total number of mosquitoes analyzed.

### Data analyses

Statistical analyses and graphical displays were completed with the R version 3.6.0. (26 April 2019) [36] and the additional package “lsmeans” [37]. Generalized binomial regression models (GLM) were used to investigate the effect of mosquito species, the days post infection, and the virus titer in the blood meal including their interactions (explanatory variables) on the feeding, survival, infection, dissemination, and transmission rates and transmission efficiencies (response variables). Least-squares means (LSM) [38] were used for testing linear contrasts among predictions with Tukey’s adjustment for *P*-values [39]. Results were considered statistically relevant when the *P*-values (summarized in Additional file 1: Tables S1–S3) were < 0.05. In the case of highly unbalanced mosquito numbers, a Fisher’s exact test with Bonferroni correction was implemented instead.

Individual samples where the extremities were virus-positive but not the bodies were not included in the analyses. Two mosquitoes had USUV-positive bodies and saliva even though USUV-specific RNA quantities in their legs plus wings were close to the detection limit. However, before virus could be secreted with the saliva, the mosquitoes must have developed a disseminated infection. Therefore, the two samples were included when counting USUV-positive legs plus wings samples, and the low viral RNA quantities in the legs plus wings were attributed to errors in the extraction process.

## Results

### Feeding and survival rates

**Fig. 1:**
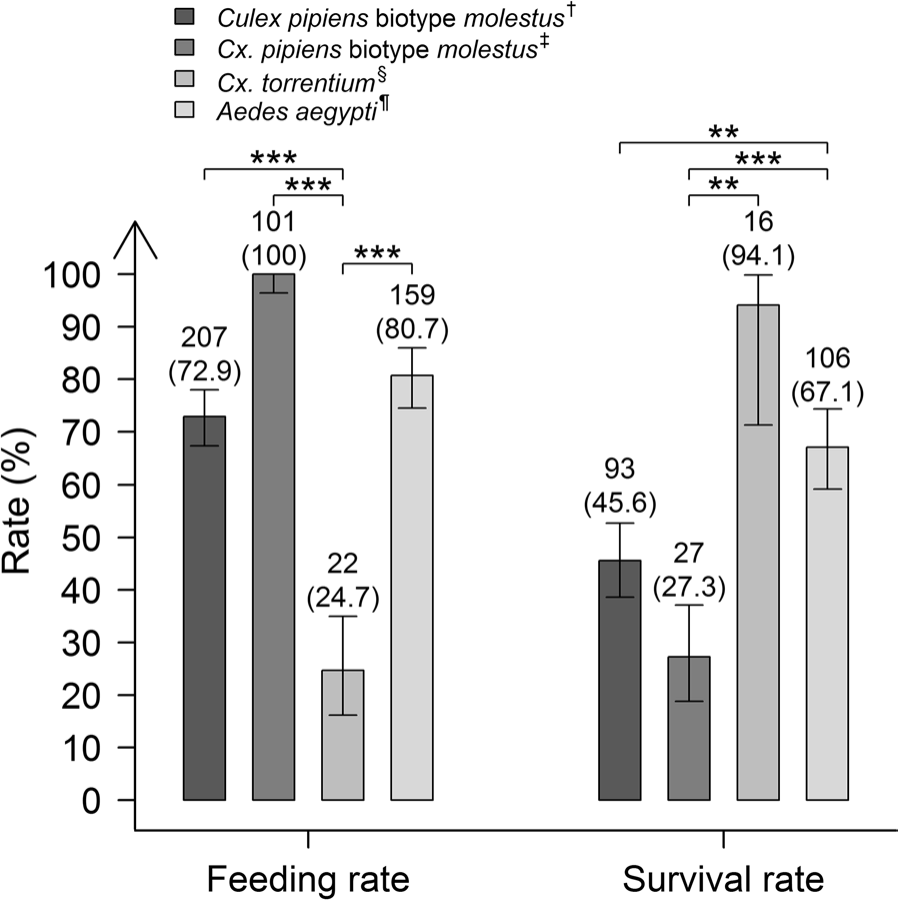
Comparison of the feeding and survival rates (from 0 to 14/16 dpi) of the four tested mosquito populations. Data values above the bars indicate the number of fully engorged or survived females per species, respectively. Numbers in brackets specify the ratio of engorged and survived females to the total number of females exposed to a blood meal or subjected to the experiment (minus day-0 samples), respectively. Error bars represent 95% confidence intervals. *P < 0.05, **P < 0.01, and ***P < 0.001 by generalized binomial regression models or Fisher’s exact test with Bonferroni correction. ^†^*Cx. pipiens* biotype *molestus* laboratory colony from “Wendland,” Lower Saxony, Germany. ^‡^*Cx. pipiens* biotype *molestus* laboratory colony from Novi Sad, the Republic of Serbia. ^§^*Cx. torrentium* field-collected colony near Berlin and Bonn, North Rhine-Westphalia, Germany. ^¶^*Ae. aegypti* laboratory colony from Malaysia (Bayer CropScience, Langenfeld, Germany)

Of the 284 German and the 101 Serbian *Cx. pipiens* biotype *molestus*, which were offered an infectious blood meal, 72.9% and 100%, respectively, were fully engorged after exposure (Fig. 1). Similarly high was the feeding rate in the *Ae. aegypti* with 80.7% of the 197 exposed females. By contrast, of the F0 generation of the field-collected *Cx. torrentium*, only 24.7% of the 89 females took a blood meal, significantly less than of the *Ae. aegypti* and both the *Cx. pipiens* biotype *molestus* laboratory colonies from Germany and Serbia (Fisher’s exact test: *P* < 0.001 for all). The few engorged *Cx. torrentium* females, however, showed high survival rates of 94.1% and 100% from 0 to 14/16 dpi and from 14/16 to 21 dpi (Fig. 1; only including the survival rates from 0 to 14/16 dpi), respectively, while survival rates of the laboratory colonies after feeding from the infectious blood meal were lower. However, differences were only significant for groups of mosquitoes surviving from 0 to 14/16 dpi but not from 14/16 to 21 dpi (summarized in Additional file 1: Table S3). For example, from 0 to 14/16 dpi, the survival rate of the German *Cx. pipiens* biotype *molestus* was 45.6%, which was significantly lower than for *Ae. aegypti* with 67.1% (GLM and LSM: *df* = infinity, *Z*-ratio =  4.0, *P* = 1.4 × 10^−3^). Similarly, the survival rate of the Serbian *Cx. pipiens* biotype *molestus* was 27.3%, which was significantly lower than the survival rates of both *Ae. aegypti* (GLM and LSM: *df* = infinity, *Z*-ratio = 6.0, *P* < 0.001) and *Cx. torrentium* (GLM and LSM: *df* = infinity, *Z*-ratio = − 3.6, *P* = 9.0 × 10^−3^).

### Infection, dissemination, and transmission rates

The infection, dissemination, and transmission rates of the four mosquito infection experiments conducted in this study are summarized in Table 1. The virus titers of the blood meals were distinguished as being either high with 10^7.4^ TCID_50_/ml or low with 10^5.1^ TCID_50_/ml. After uptake via the infectious blood meal, USUV was able to establish an infection in all of the tested species. However, only *Cx. pipiens* biotype *molestus* and *Cx. torrentium* proved competent for USUV. After oral exposure to a high virus titer (10^7.4^ TCID_50_/ml) in the blood meals, viral RNA could be detected in the bodies, legs plus wings, and saliva samples of multiple mosquitoes from both the German and Serbian *Cx. pipiens* biotype *molestus* colonies. The saliva secreted by six of the German and six of the Serbian *Cx. pipiens* biotype *molestus* mosquitoes contained virus particles that successfully infected mammalian Vero cells with substantial virus replication 7 dpi. Significant differences, however, were not observed among the vector competence indices (infection, dissemination, and transmission rates) between either the mosquito species or the collection dates (i.e. 14/16 and 21 dpi). This, however, may be a result of the small sample sizes per species and time point. By contrast, a low virus titer (10^5.1^ TCID_50_/ml) in the blood meals yielded a very low infection rate of 5.6% (2/36) 14 dpi and 5.3% (1/19) 21 dpi in the German *Cx. pipiens* biotype *molestus* with no virus replication in secondary tissue. Nonetheless, the low virus titer still infected one out of a total of eight *Cx. torrentium* specimens 21 dpi, with subsequent virus dissemination and transmission. Extensive virus replication took place with RNA viral loads of 3.9 × 10^6^ per µl of total RNA in the body and 4.7 × 10^4^ per µl of total RNA in the legs plus wings. In comparison, the day-0 *Cx. torrentium* mosquitoes contained on average 3.5 × 10^4^ per µl of total RNA. Furthermore, the virus titer in the blood meals not only affected the infection, dissemination, and transmission rates but also the viral loads in the individual mosquito samples. For example, after oral inoculation with a high titer, 6.3 × 10^5^ virus copies per µl of total RNA were found on average in the bodies and 1.2 × 10^4^ virus copies per µl of total RNA in the legs plus wings of the German *Cx. pipiens* biotype *molestus* 14/16 and 21 dpi*.* This is higher than the viral load found in the day-0 samples with 1.1 × 10^4^ virus copies per µl of total RNA. A low virus titer resulted in a decline in the number of virus copies to 8.7 × 10^1^ per µl of total RNA in the bodies and no virus copies in the legs plus wings of the German *Cx. pipiens* biotype *molestus* 14/16 and 21 dpi. Even when exposed to a high virus titer, *Ae. aegypti* had an infection rate of 0% 14/16 dpi and only 18.2% 21 dpi, with the detection of virus dissemination in only one mosquito. USUV could not be detected in the saliva of *Ae. aegypti* mosquitoes.

**Table 1:** Infection, dissemination, and transmission rates of mosquitoes infected with the German USUV Africa 2 strain

Blood meal virus titer (TCID_50_/ml)	Mosquito species	Dpi	Infection rate (%) (95% CI)	Mean viral load bodies (viral copies/µl of total RNA)	Dissemination rate (%) (95% CI)	Mean viral load legs plus wings (viral copies/µl of total RNA)	Transmission rate (%) (95% CI)
High titer 10^7.4^	*Culex pipiens* biotype *molestus*^a^	14	8/10 (80.0) (44.4–97.5)	6.9 × 10^5^	3/8 (37.5) (8.5–75.5)	9.0 × 10^3^	3/3 (100) (29.2–100)
		21	4/6 (66.7) (22.3–95.7)	5.6 × 10^5^	4/4 (100) (39.7–100)	1.5 × 10^4^	3/4 (75.0) (19.4–99.4)
	*Cx. pipiens* biotype *molestus*^b^	16	13/16 (81.3) (54.4–96.0)	1.9 × 10^6^	13/13 (100) (75.3–100)	7.8 × 10^4^	2/13 (15.4) (1.9–45.4)
		21	8/10 (80.0) (44.4–97.5)	8.1 × 10^5^	8/8 (100) (63.1–100)	7.8 × 10^4^	4/8 (50.0) (15.7–84.3)
	*Aedes aegypti*^d^	14	0/53 (0) (0–6.7)	NA	NA	NA	NA
		21	4/22 (18.2) (5.2–40.3)	2.3 × 10^5^	1/4 (25.0) (0.6–80.6)	5.5 × 10^3^	0/1 (0) (0–97.5)
Low titer 10^5.1^	*Cx. pipiens* biotype *molestus*^a^	14	2/36 (5.6) (0.7–18.7)	1.2 × 10^2^	0/2 (0) (0–84.2)	NA	NA
		21	1/19 (5.3) (0.7–18.7)	5.4 × 10^1^	0/1 (0) (0–84.2)	NA	NA
	*Cx. torrentium*^c^	14	1/8 (12.5) (0.3–52.7)	2.8 × 10^1^	0/1 (0) (0–97.5)	NA	NA
		21	1/8 (12.5) (0.3–52.7)	3.9 × 10^6^	1/1 (100) (2.5–100)	4.7 × 10^4^	1/1 (100) (2.5–100)

Transmission rates include results from the saliva inoculation on Vero cells and from the RT-qPCRs of cell culture supernatants. All mosquitoes were incubated for 14/16 or 21 days. Absolute quantification of virus copies/µl of total RNA was performed via an RT-qPCR-based calibration curve

*CI* confidence interval, *dpi* days post infection, *NA* not applicable

^a^*Cx. pipiens* biotype *molestus* laboratory colony from “Wendland,” Lower Saxony, Germany

^b^*Cx. pipiens* biotype *molestus* laboratory colony from Novi Sad, the Republic of Serbia

^c^*Cx. torrentium* field-collected colony near Berlin and Bonn, North Rhine-Westphalia, Germany

^d^*Ae. aegypti* laboratory colony from Malaysia (Bayer CropScience, Langenfeld, Germany)

## Discussion

The goal of this study was to identify German mosquito species that are vector-competent for USUV to establish evidence-based recommendations regarding the implementation of mosquito surveillance and control strategies. The study proved for the first time that Serbian and German *Cx. pipiens* biotype *molestus* and German *Cx. torrentium* mosquito populations are competent in replicating and transmitting the German USUV Africa 2 strain from Berlin. The vector competence of *Culex* mosquitoes for zoonotic viruses like USUV is, nevertheless, not a new discovery. Several infection experiments have been performed in the past with *Culex* species [6]. This genus includes species which are not only abundant in Europe but can also function as bridge vectors because of the high variability in their host-feeding patterns, transmitting zoonotic pathogens from birds to mammals [40]. A *Cx. neavei* population from Senegal, a species which is ubiquitous in Africa, was verified to be highly competent for the Africa 2 USUV strain SAAR-1776 (GenBank accession no. AY453412) and is therefore a likely endemic vector in the USUV transmission cycle in Africa. Fourteen days post exposure to a blood meal with a virus titer of 10^6.5^ plaque forming units (PFU) per ml, its infection rate was 91%, its dissemination rate 40%, and its transmission rate 81% [22]. These values are very similar to the rates found in this report for the German *Cx. pipiens* biotype *molestus*. Recently performed infection experiments with a *Cx. pipiens* population from The Netherlands [24] demonstrated for the first time the ability of European mosquitoes to transmit a Europe 2 USUV strain from Italy (Bologna/09; GenBank accession no. HM569263). Similarly to the African *Cx. neavei* and the German *Cx. pipiens* biotype *molestus* from this study, the infection rates were high (80%), with virus dissemination and accumulation rates in the saliva of 69% [24]. However, contrary to the infection of the European mosquitoes with the Italian USUV strain, two *Cx. pipiens* populations from the UK [41] showed a low susceptibility to the African SAAR-1776 strain. Infection rates ranged from 0 to 14.2%, and only 1 out of 48 *Cx. pipiens* was positive for USUV in its saliva 14 dpi [41]. The variation in susceptibility could be due to the genetic variability of the virus strains with in situ evolution and host-specific mutations in European but not African lineages [14]. Therefore, it is especially interesting that *Cx. pipiens* and *Cx. quinquefasciatus* colonies from North America [23], where USUV has not been detected yet, proved highly susceptible to the very same Africa 2 strain (SAAR-1776). *Culex pipiens* showed an infection rate of 59%, and of these, 24% were positive in their saliva. *Culex quinquefasciatus* yielded similar results [23]. It is, therefore, highly likely that varying blood meal virus titers (10^7.5^ compared to 10^6.0^ TCID_50_/ml) or disparities in the vector competence of geographically distinct mosquito populations of the same species also influenced the results.

So far, no vector competence studies have been performed with *Cx. torrentium* and USUV. This is, therefore, the first confirmation of USUV transmission by a *Cx. torrentium* specimen. Due to the limited availability of *Cx. torrentium* females for this study the vector competence of this species was only examined after feeding from a blood meal with a low virus titer. It would be interesting to also test the species’ vector competence after feeding from a high virus titer and compare it to that of other species. Only recently was a German *Cx. torrentium* population described to be highly competent for the closely related flavivirus WNV with transmission rates of up to 90% [42]. *Culex torrentium* even transmitted WNV to a greater degree than *Cx. pipiens* biotype *molestus* [42].

In addition to the results from the vector competence experiments, the role of *Culex* mosquitoes in the transmission of USUV is reinforced through the findings of USUV in native mosquitoes in Africa and Europe. For example, USUV was detected in *Cx. antennatus*, *Cx. modestus*, *Cx. neavei*, *Cx. perexiguus*, *Cx. perfuscus*, *Cx. pipiens*, and *Cx. quinquefasciatus* [6]. Since the first isolation of USUV in Germany in 2010, multiple detections in *Cx. pipiens* sensu lato (s.l.) have been described: in 2014 near Freiburg, Baden-Wuerttemberg (Europe 3) [43], in 2015 near Leipzig, Saxony-Anhalt (Africa 2) [44], and in 2016 in Emsdetten, North Rhine-Westphalia (Africa 3) [43]. To date, USUV-positive *Cx. pipiens* s.l. have also been collected in Austria, France, Italy, Serbia, Spain, and Switzerland [6]. It must, however, be kept in mind that the mere isolation of USUV from field-collected, homogenized, whole mosquitoes cannot give a precise indication on the vector status of a specific mosquito species.

Of the invasive mosquito species in Europe, USUV nucleic acid was isolated from the Asian bush mosquito *Ae. japonicus* in Graz, Austria [45], and the Asian tiger mosquito *Ae. albopictus* in Emilia-Romagna region, Italy [46–48]. Two vector competence studies have described *Ae. albopictus* as being refractory to an USUV infection, where neither an African (SAAR-1776) nor various Italian strains (GenBank accession nos. KF055442, KF055441, and KF055440) resulted in virus accumulation in the mosquitoes’ saliva [23, 28]. In comparison, this study also investigated the USUV vector competence of the yellow fever mosquito *Ae. aegypti*. This species is thermophilic and endemic in tropical and subtropical regions of the world. Multiple introductions into Europe have been described, such as into a German household [49] and a Dutch airport [50], by means of passive mosquito dispersal. Similarly to *Ae. albopictus*, the results of this study show that *Ae. aegypti* mosquitoes are relatively refractory to USUV. The species is, therefore, unlikely to be involved in the transmission of USUV in Europe despite virus replication and dissemination in individual specimens. The infection rates of both the German and Serbian *Cx. pipiens* biotype *molestus* colonies were higher than those of the tested *Ae. aegypti*. None of the tested *Ae. aegypti* saliva samples contained viable virus. Nonetheless, due to the ongoing expansion of invasive species such as *Ae. albopictus* [51, 52] and *Ae. japonicus* [53, 54], the need to also investigate the role of these mosquito species in the transmission of USUV to incidental hosts (e.g. humans and equines) remains.

Multiple studies have demonstrated the virus-dose dependency of a mosquito species’ vector competence after an oral infection with USUV [22] and WNV [55]. This correlates with the results in this report where a two-log drop in the virus titer resulted in the absence of USUV-specific RNA in secondary tissues and the saliva of German *Cx. pipiens* biotype *molestus*. The generalized binomial regression model calculated a significant increase in the infection rates after exposure to a high rather than a low virus titer in the blood meals (GLM and LSM: *df* = infinity, *Z*-ratio = − 4.7, *P* < 0.001). Nonetheless, recurring USUV outbreaks among the German avifauna, such as in 2017 and 2018 [19], verify the probable presence of numerous wild bird species producing viral loads high enough to infect susceptible mosquito species. In the case of the closely related WNV, viremia levels in birds can reach 10^12^ TCID_50_/ml, yet most birds do not reach titers > 10^8^ TCID_50_/ml [55].

The virus titer is not the only variable to take into account, as there is also a strong temperature dependency of vector competence. For example, the Dutch *Cx. pipiens* infected with the Italian USUV strain had distinctly higher infection rates at 28 °C than at 18 °C (90% versus 11%) [24]. This temperature dependency of vector competence is multifactorial and varies between mosquito species. The positive correlation between the vector competence for USUV and the ambient temperature is among others a result of the ectothermic nature of mosquitoes, where an increase in temperature directly results in an increase in the replication rate of the virus [56]. This, in turn, results in a reduction of the extrinsic incubation period [56–58]. Furthermore, a reduced effectiveness of the midgut barrier at higher temperatures, temperature-induced changes in the regulation of biotype-specific immune-responsive genes, and temperature-dependent activation of RNA interference pathways can all influence vector competence [58]. All experiments in this study were performed at 25 °C. This temperature resembles the average summer conditions around the Mediterranean Sea and southeastern European countries but is higher than the recorded long term mean temperatures of German summers. Yet, extreme heat waves such as in 2003, 2018, and 2019 have clearly demonstrated that an increase from the long-term mean temperatures in Germany is becoming more frequent [59, 60]. The summer of 2018 was the second warmest ever recorded in Germany, possibly shortening the extrinsic incubation period and allowing a more rapid autochthonous virus transmission [61]. Therefore, the occurrence of temperatures high enough to support the USUV vector-host transmission cycle in Germany is also conceivable and can help explain the increasing frequency of avian infections [18, 19, 62].

In this experiment, numerous other factors may have had an effect on the results. Aside from the *Cx. torrentium* specimens, all mosquitoes originated from established colonies with multiple generations in the laboratory. This may have had an impact on the genetic and phenotypic variations of the mosquito strains. The susceptibility of a mosquito to a virus cannot only be generation- but also time- and population-specific, with geographic variations in the vector competence [55, 63]. Furthermore, to obtain optimal feeding rates a sugar solution was applied to the blood meals. Even though a final sugar concentration of 1.5–1.8% in the blood meals is probably too low to influence a mosquito’s vector competence [64], its influence on the mosquito’s immune response and its gut microbiota [64, 65] and therefore also on its vector competence for USUV remains unclear. Not only can the gut microbiome or the presence of intracellular *Wolbachia* bacteria influence a mosquito’s vector competence but also coinfections with other viruses or pathogens. For example, the German and Serbian *Cx. pipiens* biotype *molestus* colonies tested competent for USUV in this study already proved their high transmission efficiencies for a German WNV isolate [66]. USUV and WNV coinfections are not only highly conceivable in mosquitoes but have also been described in 34 avian species and in horses in Europe [7]. Future studies should, therefore, focus on what effect viral interferences will have on the antibody production in susceptible hosts and the immune response of mosquitoes [67].

## Conclusion

Both the German (“Central European”) and Serbian (“Southern European”) *Cx. pipiens* biotype *molestus* laboratory colonies could transmit the USUV Africa 2 lineage at an extrinsic incubation temperature of 25 °C. A specimen obtained from field-collected egg rafts of *Cx. torrentium*, a highly prevalent species in Northern Europe, was also capable of transmitting USUV. The tropical mosquito species *Ae. aegypti*, on the other hand, showed reduced susceptibility with no detection of virus in its saliva.

## Supplementary Information

**Additional file 1: Table S1**. Overview of explanatory variables included in the final generalized binomial regression models for the investigated rates at 25 °C. **Table S2.** *P*-values of the fixed effects in the least-square means analysis when comparing the rates between all species at 25 °C in the final generalized binomial regression models specified in Table S1. *P*-value adjustment was performed using the Tukey method. **Table S3.** *P*-values of the interaction term in the least-square means analysis for the survival, infection, and transmission rates at 25 °C.

## Data Availability

Data supporting the conclusions of this article are included within the article and its additional files. Raw data generated and/or analyzed during the present study are not publicly available, but are available from the corresponding author upon reasonable request.

## Abbreviations

CI: Confidence interval
CO_2_: Carbon dioxide
dpi: Days post infection
FCS: Fetal calf serum
FLI: Friedrich-Loeffler-Institut
GLM: Generalized binomial regression model
LSM: Least-squares mean
MEM: Minimum essential medium
NA: Not applicable
PBS: Phosphate-buffered saline
PCR: Polymerase chain reaction
PFU: Plaque forming units
RNA: Ribonucleic acid
RT-qPCR: Reverse transcription quantitative real-time PCR
s.l.: Sensu lato
TCID_50_: Tissue culture infective dose
UK: The United Kingdom
USA: The United States of America
USUV: Usutu virus
WNV: West Nile virus
