# Supplementary material for: German Culex pipiens biotype molestus and Culex torrentium are vector-competent for Usutu virus
Source: Parasit Vectors. 2020 Dec 30;13:625. doi: 10.1186/s13071-020-04532-1 (PMC7774236; doi:10.1186/s13071-020-04532-1)
Supplement: Supplementary file 1 — Additional file 1: Table S1. Overview of explanatory variables included in the final generalized binomial regression models for the investigated rates at 25 °C. Table S2. P-values of the fixed effects in the least-square means analysis when comparing the rates between all species at 25 °C in the final generalized binomial regression models specified in Table S1. P-value adjustment was performed using the Tukey method. Table S3. P-values of the interaction term in the least-square means analysis for the survival, infection, and transmission rates at 25 °C. [file 13071_2020_4532_MOESM1_ESM.docx]

**Additional file 1: Statistical analyses**

**Table S1.** Overview of explanatory variables included in the final generalized binomial regression models for the investigated rates at 25 °C.

|  | **Response variables** | | | | | |
| --- | --- | --- | --- | --- | --- | --- |
| **Explanatory variables** | **Feeding**  **rate** | **Survival**  **rate** | **Infection**  **rate** | **Dissemination rate** | **Transmission rate** | **Transmission efficiency** |
| **Species** | x | x | x | x | x | x |
| **Days post infection** | / | x | x | x | x | x |
| **Interaction term: Species: days post infection** | / | x | x | / | x | / |
| **Interaction term:**  **Species: blood meal titer** | / | / | x | / | / | / |

**Table S2.** *P*-values of the fixed effects in the least-square means analyses when comparing the rates between all species at 25 °C in the final generalized binomial regression models (GLM) specified in Table S1. *P*-value adjustment was performed using the Tukey method.

| **Fixed effects:** | |  |  |  |  |  |  |  |
| --- | --- | --- | --- | --- | --- | --- | --- | --- |
|  | | **Feeding**  **rate** | | **Survival**  **rate^+^** | **Infection**  **rate^+^** | **Dissemination rate** | **Transmission rate^+^** | **Transmission efficiency** |
|  | **Contrast** | ***P*.value**  **(GLM)** | ***P*.adjusted (Fisher)^‡^** | ***P*.value** | ***P*.value** | ***P*.value** | ***P*.value** | ***P*.value** |
| **Species** | AA_M - CPM_G | 1.99 x 10^-1^ | 1.00 | 7.62 x 10^-1^ | 1.00 | 3.26 x 10^-1^ | NA | 1.00 |
|  | AA_M - CPM_S | 1.00 | 1.00 | 8.45 x 10^-1^ | 1.00 | 1.00 | NA | 1.00 |
|  | AA_M - CT_G | 4.29 x 10^-14^* | 1.30 x 10^-5^* | 1.00 | 1.00 | 6.86 x 10^-1^ | NA | 1.00 |
|  | CPM_G - CPM_S | 1.00 | 3.86 x 10^-1^ | 6.12 x 10^-1^ | 9.64 x 10^-1^ | 1.00 | 1.00 | 7.21 x 10^-1^ |
|  | CPM_G - CT_G | 3.57 x 10^-13^* | 4.58 x 10^-5^* | 1.00 | 7.48 x 10^-1^ | 9.97 x 10^-1^ | NA | 3.18 x 10^-1^ |
|  | CPM_S - CT_G | 1.00 | 8.65 x 10^-7^* | 1.00 | 9.60 x 10^-1^ | 1.00 | NA | 6.58 x 10^-1^ |
| **Days post infection** | 14/16 - 21 | NA | NA | 9.76 x 10^-1^ | 9.91 x 10^-1^ | 4.50 x 10^-2^* | NA | 8.95 x 10^-2^ |
| **Blood meal virus titer (TCID_50_/mL)** | 10^5.1-7.4^ | NA | NA | NA | 2.11 x 10^-6^* | NA | NA | NA |

* *P* < 0.05.

^+^ Results for fixed effects may be misleading due to involvement in interactions (Table S3).

^‡^ Additional statistical evaluation with Fisher’s exact test with Bonferroni correction.

NA: not applicable.

AA_M: *Aedes aegypti* from Malaysia.

CPM_G: *Culex pipiens* biotype *molestus* from Germany.

CPM_S: *Cx. pipiens* biotype *molestus* from the Republic of Serbia.

CT_G: *Cx. torrentium* from Germany.

**Table S3.** *P*-values of the interaction term in the least-square means analyses for the survival, infection, and transmission rates at 25 °C.

| **Interactions:** | |  |  |  |
| --- | --- | --- | --- | --- |
|  | | **Survival rate** | **Infection**  **rate** | **Transmission rate** |
| **Species: days post infection** | **Contrast** | ***P*.value** | ***P*.value** | ***P*.value** |
|  | AA_M,14 - CPM_G,14 | 1.38 x 10^-3^* | 1.00 | NA |
|  | AA_M,14 - CPM_S,16 | 5.47 x 10^-8^* | 1.00 | NA |
|  | AA_M,14 - CT_G,14 | 5.01 x 10^-1^ | 1.00 | NA |
|  | AA_M,14 - AA_M,21 | 3.84 x 10^-2^* | 1.00 | NA |
|  | AA_M,14 - CPM_G,21 | 6.68 x 10^-1^ | 1.00 | NA |
|  | AA_M,14 - CPM_S,21 | 8.09 x 10^-1^ | 1.00 | NA |
|  | AA_M,14 - CT_G,21 | 1.00 | 1.00 | NA |
|  | CPM_G,14 - CPM_S,16 | 5.11 x 10^-2^ | 1.00 | 1.00 |
|  | CPM_G,14 - CT_G,14 | 8.65 x 10^-2^ | 9.99 x 10^-1^ | NA |
|  | CPM_G,14 - AA_M,21 | 1.00 | 3.72 x 10^-2^* | 1.00 |
|  | CPM_G,14 - CPM_G,21 | 9.82 x 10^-1^ | 1.00 | 1.00 |
|  | CPM_G,14 - CPM_S,21 | 2.70 x 10^-1^ | 1.00 | 1.00 |
|  | CPM_G,14 - CT_G,21 | 1.00 | 9.99 x 10^-1^ | 1.00 |
|  | CPM_S,16 - CT_G,15 | 8.96 x 10^-3^* | 1.00 | NA |
|  | CPM_S,16 - AA_M,21 | 5.77 x 10^-1^ | 1.06 x 10^-2^* | 1.00 |
|  | CPM_S,16 - CPM_G,21 | 5.44 x 10^-2^ | 9.99 x 10^-1^ | 4.68 x 10^-1^ |
|  | CPM_S,16 - CPM_S,21 | 4.58 x 10^-2^* | 1.00 | 7.31 x 10^-1^ |
|  | CPM_S,16 - CT_G,21 | 1.00 | 1.00 | 1.00 |
|  | CT_G,14 - AA_M,21 | 7.55 x 10^-2^ | 2.33 x 10^-1^ | NA |
|  | CT_G,14 - CPM_G,21 | 2.09 x 10^-1^ | 9.89 x 10^-1^ | NA |
|  | CT_G,14 - CPM_S,21 | 1.00 | 1.00 | NA |
|  | CT_G,14 - CT_G,21 | 1.00 | 1.00 | NA |
|  | AA_M,21 - CPM_G,21 | 9.61 x 10^-1^ | 2.03 x 10^-1^ | 1.00 |
|  | AA_M,21 - CPM_S,21 | 2.38 x 10^-1^ | 5.53 x 10^-2^ | 1.00 |
|  | AA_M,21 - CT_G,21 | 1.00 | 2.33 x 10^-1^ | 1.00 |
|  | CPM_G,21 - CPM_S,21 | 4.84 x 10^-1^ | 1.00 | 9.93 x 10^-1^ |
|  | CPM_G,21 - CT_G,21 | 1.00 | 9.89 x 10^-1^ | 1.00 |
|  | CPM_S,21 - CT_G,21 | 1.00 | 1.00 | 1.00 |

* *P* < 0.05.

NA: not applicable.

AA_M: *Aedes aegypti* from Malaysia.

CPM_G: *Culex pipiens* biotype *molestus* from Germany.

CPM_S: *Cx. pipiens* biotype *molestus* from the Republic of Serbia.

CT_G: *Cx. torrentium* from Germany.
